# Formulation of Sugar/Hydrogel Inks for Rapid Thermal Response 4D Architectures with Sugar-derived Macropores

**DOI:** 10.1038/s41598-020-64457-8

**Published:** 2020-05-05

**Authors:** Hyojin Ko, Monica Cahyaning Ratri, Kihoon Kim, Yeongheon Jung, Giyoong Tae, Kwanwoo Shin

**Affiliations:** 1 Department of Chemistry and Institute of Biological Interfaces, Sogang University, Seoul, 04107 Republic of Korea; 2 Department of Chemistry Education, Sanata Dharma University, Yogyakarta, 55281 Republic of Indonesia; 3 School of Materials Science and Engineering, Gwangju Institute of Science and Technology, Gwangju, 61005 Republic of Korea

**Keywords:** Bioinspired materials, Gels and hydrogels, Polymers

## Abstract

Programmed, reshaping hydrogel architectures were fabricated from sugar/hydrogel inks via a three-dimensional printing method involving a stimuli-responsive polymer. We developed a new hydrogel ink composed of monomers (acrylamide [AAm]) and N-isopropylacrylamide [NIPAAm]), and sugar (mixture of glucose and sucrose) as a pore-generator, enabling to improve printability by increasing the ink’s viscoelastic properties and induce the formation of macropores in the hydrogel architectures. This study demonstrated that creating macropores in such architectures enables rapid responses to stimuli that can facilitate four-dimensional printing. We printed bilayer structures from monomer inks to which we had added sugar, and we exposed them to processes that cross-linked the monomers and leached out the sugar to create macropores. In comparison with a conventional poly(N-isopropylacrylamide) hydrogel, the macroporous hydrogels prepared using polymerization in the presence of a high concentration of sugar showed higher swelling ratios and exhibited much faster response rates to temperature changes. We used rheometry and scanning electron microscopy to characterize the properties of these inks and hydrogels. The results suggest that this method may provide a readily available route to the rapid design and fabrication of shape-morphing hydrogel architectures with potential application in soft robotics, hydrogel actuators, and tissue engineering.

## Introduction

Three-dimensional (3D) printing of soft materials, such as hydrogels, elastomers, and conductive polymer composites, is an attractive approach in materials science, tissue engineering, soft robotics, and flexible electronics^1–6^. Structures 3D printed from stimuli-responsive hydrogels can undergo reversible volume-phase transitions in response to environmental changes, such as changes in temperature, pH, electric field, and light, making these materials highly interesting research targets^7–11^. By taking advantages of the reversible and repeatable mechanical movements induced by abrupt volume changes in hydrogels in response to external stimuli, one can use stimuli-responsive hydrogels to control the mechanical actions of soft actuators. Shape morphing of 3D printed structures in response to an external stimulus is called four-dimensional (4D) printing, where time is considered as the fourth dimension. Four-dimensional printing utilizing stimuli-responsive materials and advanced 3D printing strategies is the next-generation fabrication technology^12–15^, aiming to create dynamic 3D structures capable of transforming shape or behavior in response to stimuli in a manner that mimics the movements and structures in nature, such as nastic motions in plants^16^.

Current research in 4D printing mainly focuses on (1) the development of novel materials that are able to respond to various stimuli^17^, and (2) the implementation of various shape memory geometries through physical designs for more applications^18^. However, if the gripping motions of soft robotics machines^19^ or the snapping motions of the Venus flytrap leaf (<100 ms)^16^ are to be realized, a highly precise rapidity is a core property. In this regard, a fast rate of volume change, particularly for stimuli-responsive hydrogels which undergo volume expansion upon hydration, is vital for the widespread application of 4D printing^20^. However, most polymer actuators suffer from relatively slow and small-scale movements^7–9,12,13^, and they involve complex preparation procedures, such as multistep lithography. The ability to manufacture polymer actuators by using 3D printing would enable easy fabrication and programmable shape morphing by design. Motivated by previous studies^21–26^ of the enhanced swelling and shrinking rates of hydrogels with macroporous architectures, we have designed a process that prepares hydrogel architectures containing macropores (i.e., pores larger than 10 μm) to overcome slow actuation kinetics. Sugar has been used as a porogen to induce macroporosity^22,23,27^. Adding sugar to hydrogels not only results in macropores but also can modulate the viscoelastic properties of a hydrogel ink, making it suitable for 3D printing.

Among the various 3D printing methods (e.g., inkjet printing, laser-assisted printing, and microextrusion), direct ink writing is an accessible approach that is adaptable to a range of materials and that balances printer cost and printing quality^3,6^. The rheology of the ink is the most important factor for manufacturing 3D structures via direct writing. However, structures printed with existing hydrogel inks have soft and fragile mechanical properties, requiring them to be strengthened via the incorporation of an extra supporting layer (e.g., polycaprolactone), additives (e.g., nanoclay or nanocelluose), or interpenetrating polymer networks (IPNs) to increase viscoelasticity^1,2,12,28^. Single component hydrogels, such as native PNIPAAm hydrogel^29^ and alginate hydrogel^30^ have been reported, and they are physically weak, being in the range of ~10 kPa. By incorporating alginate hydrogel into a PNIPAAm hydrogel matrix, however, the mechanical strength can be increased by orders of magnitude; for example, the tensile strengths for alginate/PNIPAAm^29^ and alginate/PAAm^31^ hydrogels increased to 120 kPa and143 kPa, respectively.

In this study we created programmed, reshaping hydrogel architectures by direct write assembly and investigated their swelling characteristics. Toward this objective, we developed a new hydrogel ink composed of monomers (acrylamide [AAm]) and *N*-isopropylacrylamide [NIPAAm]), a cross-linker, a photoinitiator, sodium alginate, sugar, and water as a solvent. We hypothesized that the addition of sugar to the ink would enable us to (1) improve printability by increasing the ink’s viscoelastic properties and (2) induce the formation of macropores to enable large deformation and fast responses in the printed structures.

## Results and Discussion

**Figure 1:**
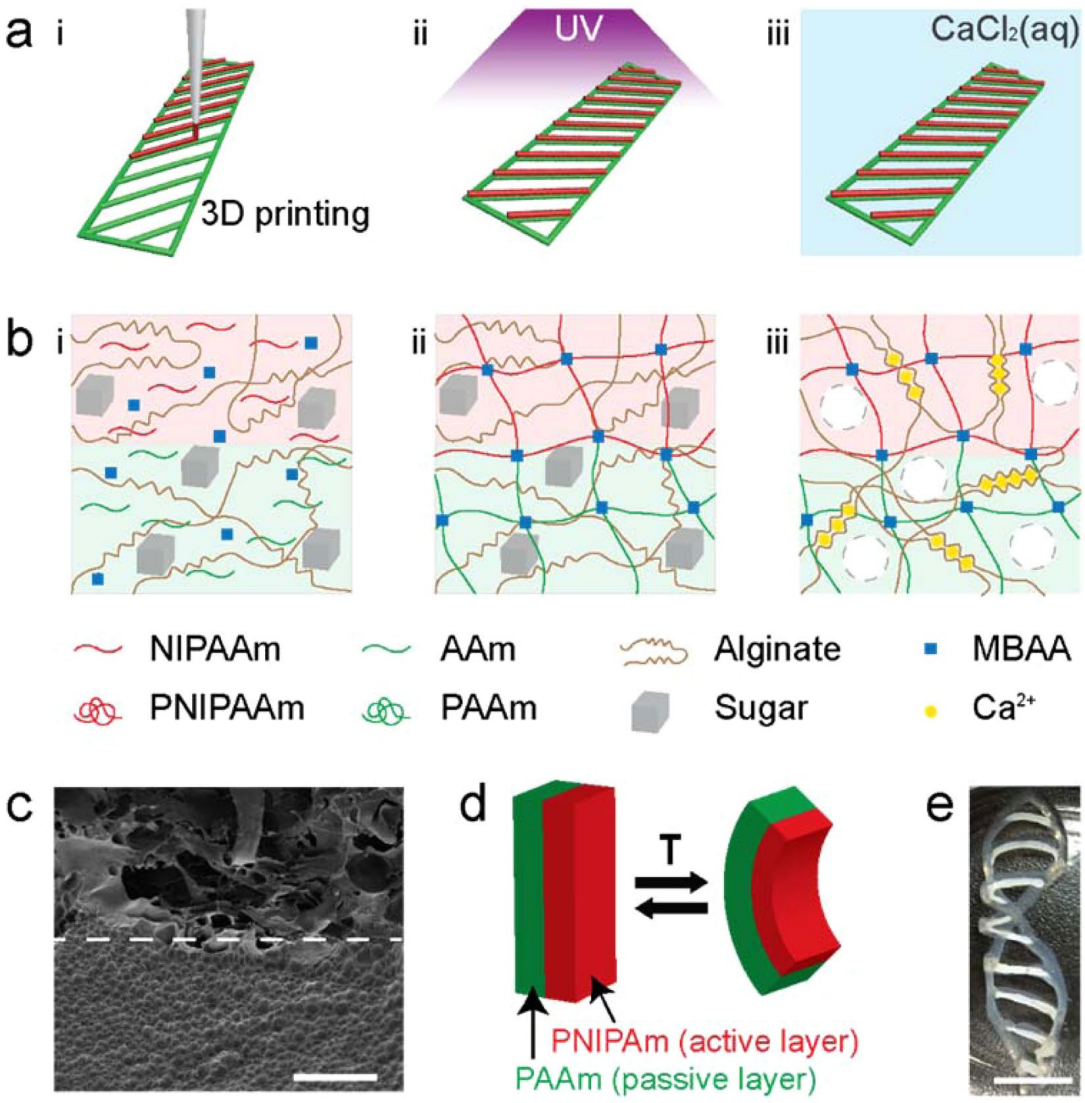
Method for printing and polymerizing macroporous hydrogels and their stimuli-responsive behavior. (**a**) Schematic of the 3D printing technique involving two cross-linking steps: (i) Three-dimensional printing of a bilayer structure with sugar/hydrogel ink, where green indicates the passive PAAm layer and red the active PNIPAAm layer; (ii) exposure to UV light to initiate polymerization and chemical cross-linking; (iii) exposure to calcium ions to initiate physical cross-linking of alginate. (**b**) Procedure for synthesizing bilayer macroporous hydrogels. (i) Prior to UV treatment, the sugar/hydrogel inks contain unlinked NIPAAm, AAm, alginate, a photoinitiator, and sugar. (ii) UV treatment induces chemical polymerization and chemical bonding between layers. (iii) Treatment with a CaCl_2_ solution induces simultaneous physical cross-linking of alginate and leaching of sugar, producing macropores inside the hydrogels. (**c**) Cross-sectional SEM image of a printed bilayer structure. The PNIPAAm layer is above the dashed line, and the PAAm layer is below it. (**d**) Reversible temperature-induced bending motion of a PNIPAAm/PAAm bilayer. T stands for temperature. (**e**) Shape morphing of a printed bilayer structure from a flat strip into a helix at high temperatures. Scale bars are 100 μm for (**c**) and 1 cm for (**e**).

We used the following process to direct write a hydrogel architecture by using this new hydrogel ink. A bilayer containing AAm and NIPAAm was printed from the new ink and immediately UV-photopolymerized with an initiator to synthesize poly(*N*-isopropylacrylamide) (PNIPAAm) and polyacrylamide (PAAm) hydrogels and to form chemical bonds between the bilayer. The polymerized structure was then immersed in calcium-chloride (CaCl_2_) solution, resulting in calcium ion–induced fast cross-linking of alginate chains in both layers and the leaching of sugar molecules to create macropores (Fig. 1a–c). The addition of alginate chains was intended not only to increase the viscosity of the ink but also to enable a double-network hydrogel in the printed structure after ionic cross-linking to improve its mechanical strength and toughness^7,32^.

The programmed, reshaping principle of hydrogel actuators is inspired by the actuating behavior of pine cones, wheat awns, and the Venus flytrap, all of which are composed of multilayered materials with different swelling/shrinking properties^9,10,15^. A bilayer consisting of two kinds of hydrogels can bend in different directions, depending on the swelling/shrinking properties of each polymer. PNIPAAm is a typical temperature-sensitive polymer that shrinks at temperatures above a lower critical solution temperature (LCST) of ~32 °C whereas PAAm exhibits no substantial change^7^. Immersion in warm water leads to contraction of the thermoresponsive PNIPAAm and deformation of the bilayer, causing it to bend (Fig. 1d). This asymmetric contraction induced by a change in the external environment is the driving force behind the bilayer’s bending motion^9^. Fig. 1e demonstrates shape change in a PNIPAAm/PAAm bilayer structure that has been immersed in warm water at 50 °C.

**Figure 2:**
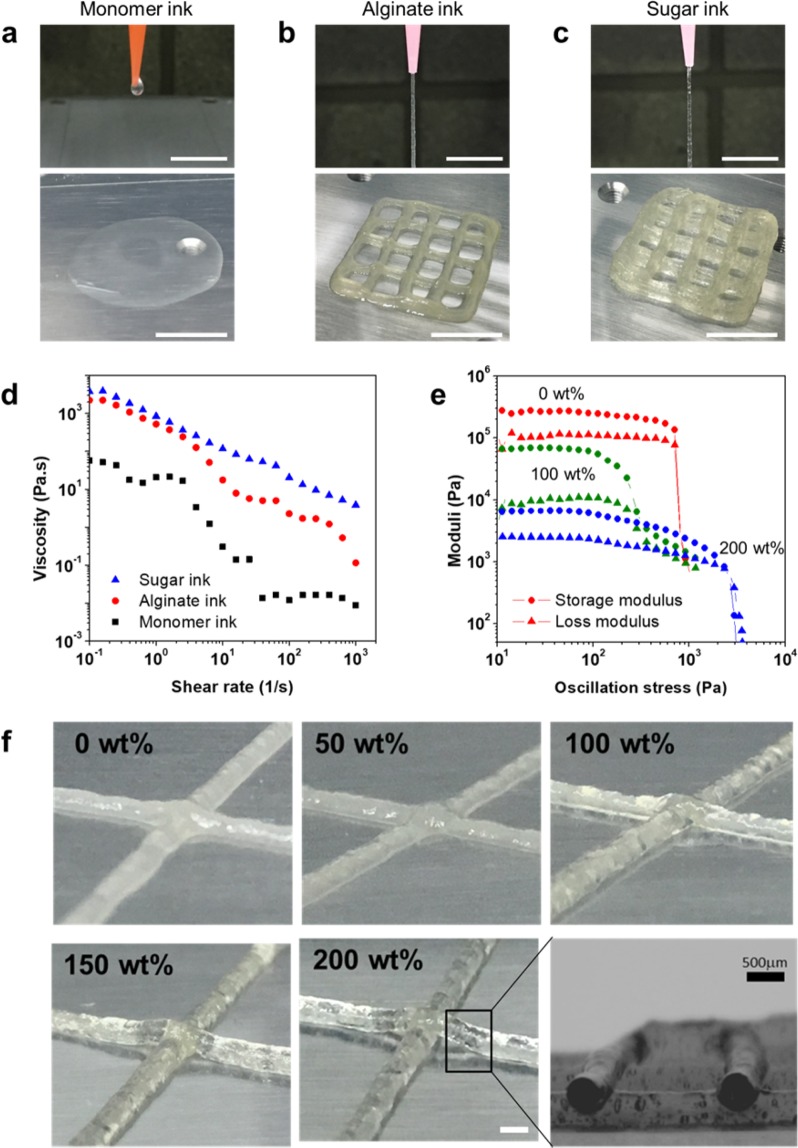
Three-dimensional (3D) printing with monomer ink, alginate ink (0 wt% sucrose), and sugar ink (200 wt% sucrose). (**a**) The monomer ink formed droplets at the nozzle and spread out on the surface. (**b,c**) The alginate ink and the sugar ink formed cylindrical shapes at the nozzle and could be printed in multilayers; however, the alginate ink collapsed, whereas the sugar ink maintained its printed shape. (**d**) Plot of the apparent viscosity as a function of the shear rate for the three inks. (**e**) Storage and loss moduli as functions of oscillation stress for different sucrose concentrations. (**f**) The shape of the intersections in the printed patterns can be seen to depend on the sucrose concentration in the ink. The inset shows a cross section of the structure printed with the sugar ink containing 200 wt% sucrose. The wt% values in (**e,f**) are sucrose concentrations with respect to the solvent. Scale bars are 1 cm for (**a–c**) and 1 mm for (**f**).

The ink’s viscosity directly influences shape fidelity after deposition. For direct ink writing, an ink viscosity of 10^2^–10^6^ mPa·s (depending on the shear rate) is required^3^. An aqueous solution of monomers, a cross-linker, and a photoinitiator was not printable because its viscosity was too low. This ink formed droplets at the nozzle and spread out on the substrate’s surface, failing to form a pattern (Fig. 2a). However, the addition of alginate increased the viscosity (>4,500% at 10^−1^ s^−1^), sufficient to enable the ink to form a cylindrical shape rather than a droplet at the nozzle, so that it could be printed as a pattern (Fig. 2b). Alginate is a typical high-viscosity material that exhibits a strong shear-thinning behavior; both characteristics are important for 3D printing^25^. A liquid phase in ink is required for effective extrusion whereas a gel/solid phase is required to maintain the as-printed structure. High-molecular-weight polymeric materials that exhibit shear-thinning behavior, such as sodium alginate, decrease in viscosity with increasing shear rate secondary to shear-induced reorganization of the polymer chains from an entangled to a more stretched conformation. After the alginate ink had been deposited onto the substrate, however, the stacked layer collapsed, and the printed layers of the ink could not maintain a cylindrical shape. The addition of sugar to the alginate ink enabled the printed structure to maintain its shape without extra support (Fig. 2c).

To facilitate printing, inks must exhibit substantial shear thinning to allow them to be extruded through fine nozzles while simultaneously exhibiting a sufficiently large storage modulus, *G*′, and yield stress, *τ*_y_, to retain both their filamentary shape upon exiting the nozzle and their shape after printing. The yield stress is the minimum stress to be applied for deformation and flow. Whereas high viscosity only delays the collapse of a deposited 3D structure, the presence of yield stress can potentially prevent flow and collapse^33^. The rheological properties of different inks are shown in Fig. 2d,e. Upon the addition of alginate, the ink’s viscosity increased compared with that of the monomer ink. The alginate ink exhibited strong shear-thinning behavior, as evidenced by the decrease in its apparent viscosity from 2500 to 0.1 Pa·s as the shear rate increased from 10^−1^ to 10^3^ s^−1^. This nearly four-fold decrease in viscosity with increasing shear rate was very similar to the value, obtained in the suitably printable range in the shear-thinning properties with hydrogels^34^. Its plateau *G*′ value was high (~2 × 10^5^ Pa), but its *τ*_y_ was rather low (~800 Pa), which seemed to result in the collapse of the stacked layers of the printed structure.

Adding sugar to the alginate ink changed the rheological properties of the ink. The sugar/hydrogel ink with 200 wt% sucrose (the percentage by weight of sugar indicates the weight of sugar relative to the deionized water used as a solvent) showed a zero shear rate viscosity and a shear-thinning behavior similar to that of the alginate gel (Fig. 2d), but was able to sustain the printed layered architectures. The plateau *G*′ value of the ink unexpectedly decreased from ~2 × 10^5^ Pa for 0 wt% (alginate ink) to ~7 × 10^4^ Pa for 100 wt% sucrose and ~6.5 × 10^3^ Pa for 200 wt% sucrose. In contrast, the ink’s yield stress increased dramatically from ~800 Pa for 0 wt% sucrose to ~1300 Pa for 100 wt% sucrose and ~2500 Pa for 200 wt% sucrose, which provided enough physical support to prevent collapse of the printed structure.

The printing fidelity of the sugar/hydrogel ink varied with its sucrose concentration (Fig. 2f). The ink with the lowest sucrose concentration exhibited a smaller yield stress and lost its cylindrical shape immediately after extrusion. Printing fidelity improved dramatically with increasing sucrose concentration owing to the higher yield stress, which prevented flow and collapse. The extruded 200 wt% ink maintained a cylindrical shape, as shown in the inset (a cross-sectioned image) to the 200 wt% image.

Glucose was also added to the sugar/hydrogel ink to modulate its rheological properties further. Compared to the ink with 200 wt% sucrose, the ink with 200 wt% glucose showed a dramatic increase in modulus to ~5 × 10^5^ Pa; however, its yield stress decreased to ~200 Pa (Fig. S1). The final composition of the sugar/hydrogel ink for 3D printing was set to 200 wt% of sucrose with 60 wt% of glucose to ensure both an adequate storage modulus and sufficient yield stress.

**Figure 3:**
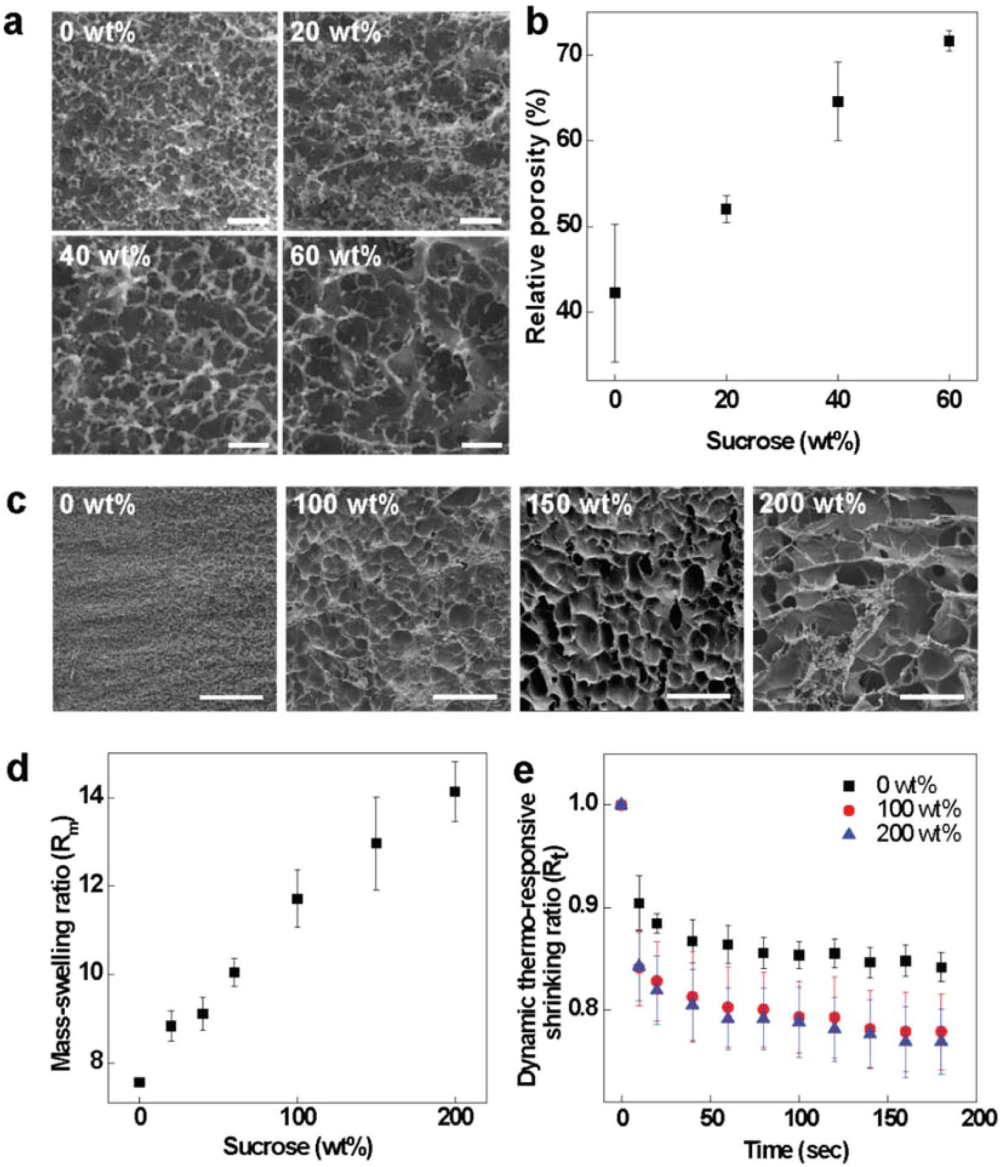
Morphology and physical properties of PNIPAAm prepared with 1-M NIPAAm and various sucrose concentrations. **(a**) SEM images showing the effect of sucrose concentration on the micropore size. (**b**) Relative porosity of hydrogels as a function of sucrose wt%. (**c**) SEM images of PNIPAAm hydrogels with higher sucrose concentrations. (**d**) Mass swelling ratio of the PNIPAAm hydrogel. (**e**) Dynamic thermoresponsive shrinking ratio (*R*_*t*_)_25→50_ of the hydrogels. Scale bars are 5 μm for (**a**) and 50 μm for (**c**). Error bars represent the standard deviations of 7 experiments for Figs. 3b and 5 experiments for Figure for 3d and e.

Increasing the amount of sugar in the ink modified not only the rheology of the ink but also the physical properties of the fabricated hydrogels (Fig. 3). A series of macroporous PNIPAAm hydrogels were prepared using solutions with various concentrations of sucrose. In terms of morphology, the conventional PNIPAAm hydrogel (0 wt%) exhibited a dense surface and small micropores. With increasing sucrose concentration, while maintaining 60 wt% of glucose, the hydrogels became more porous (Fig. 3a), and the relative porosity of the freeze-dried samples increased linearly with increasing sucrose concentration (Fig. 3b). A relatively homogeneous distribution of micropores was observed in hydrogels using formulations with less than 100 wt% sucrose. On the other hand, with 100 wt% to 200 wt% sucrose, the pore size of the hydrogels dramatically increased, presumably due to the sucrose crystals (Fig. 3c). Thus, the addition of sucrose strongly affected the pore structure of the resulting hydrogels.

As the cross-linking of alginate induced by calcium ions and the macropore formation by dissolution of sugar occur simultaneously under the same condition, it is necessary to consider how each process affects each other. According to the literatures^35,36^, the diffusion coefficient of calcium ions (~13.12 × 10^−10^ m^2^/s)^34^ is known to be 2-3 times faster than that of sucrose in water (~5.15 × 10^−10^ m^2^/s)^35^. Therefore, the crosslinking of alginate is expected to occur first, followed by the removal of sugar. As a result, the presence of sugar in the 3D structure made with this ink is considered to play an important role in determining the shape of the pores during the formation of additional double-network hydrogels.

The mass swelling ratio (*R*_*m*_) is one of the important parameters for evaluating hydrogels^37^. Fig. 3d shows the *R*_*m*_ values calculated for PNIPAAm hydrogels prepared with sucrose concentrations ranging from 0 wt% to 200 wt% with a fixed glucose concentration of 60 wt%, according to the following formula:

1 $${R}_{m}=\frac{{M}_{w}}{{M}_{d}},$$

where *M*_*w*_ is the mass of the fully swollen hydrogel in deionized water and *M*_*d*_ is the mass of the dry hydrogel. *R*_*m*_ increases with increasing sucrose concentration. We speculate that this improved swelling property results from the highly porous structure of the gels, which would induce more water absorption into the gel networks.

Compared with the conventional PNIPAAm hydrogel, the macroporous hydrogels not only had higher swelling ratios but also exhibited much faster response rates during shape morphing. To investigate how the shrinkage dynamics of the hydrogels were affected by macroporosity, we defined the dynamic thermoresponsive shrinking ratio (*R*_*t*_) as follows:

2 $${({R}_{t})}_{25\to 50}=\frac{{A}_{t}}{{A}_{0}},$$

where *A*_*t*_ is the area (cm^2^) of the hydrogel at *t* seconds after increasing temperature from 25 °C to 50 °C and *A*_0_ is the area (cm^2^) of the hydrogel at 0 s^21^. Approximately 1-mm-thick PNIPAAm hydrogels prepared in 35-mm round Petri dishes were used. After the hydrogel had been equilibrated by immersing it in deionized water at 25 °C for at least 24 h, the sample was transferred to a 50 °C bath. Compared with the conventional hydrogel (0 wt%), the hydrogels prepared with 100 wt% and 200 wt% sucrose (with a fixed glucose concentration of 60 wt%) showed faster and larger shrinking dynamics (Fig. 3e). This enhanced thermosensitivity may be important for applications such as soft actuators and robotics.

Thermosensitive shrinkage of the PNIPAAm hydrogel by heating it temperatures above the LCST can be explained by expulsion of the water molecules surrounding –CH(CH_3_)_2_ groups owing to their hydrophobicity^38^. The gel network starts to shrink, which squeezes water molecules out of the gel matrix. This reaction occurs from the outer surface of the gel, so the water molecules inside the conventional gel cannot be squeezed out immediately. In contrast, macroporous hydrogels prepared with high concentrations of sugars provide sufficient water-releasing channels for inner water molecules to be easily squeezed out of the gel matrix during transition, resulting in a much faster rate of shrinking. In addition to a fast response rate, hydrogels with large swelling ratios are also preferred. For instance, as smart actuators, hydrogels with larger swelling ratios exhibit higher degrees of shape change than those with smaller swelling ratios do; this extends the actuating performance^17^. The presence of numerous interconnected hollow spaces in macroporous gels with an open-celled structure can promote shrinkage to a compact state (Fig. S2). The results in Fig. 3 show that macroporous PNIPAAm hydrogels fabricated using our method exhibit both a large swelling ratio and a fast response rate. This approach to forming macropores inside hydrogels can easily be adapted to other polymer hydrogels. Large-amplitude shape morphing and robust responsiveness, coupled with large-scale displacement, can be attained in this way for stimuli-responsive hydrogels.

**Figure 4:**
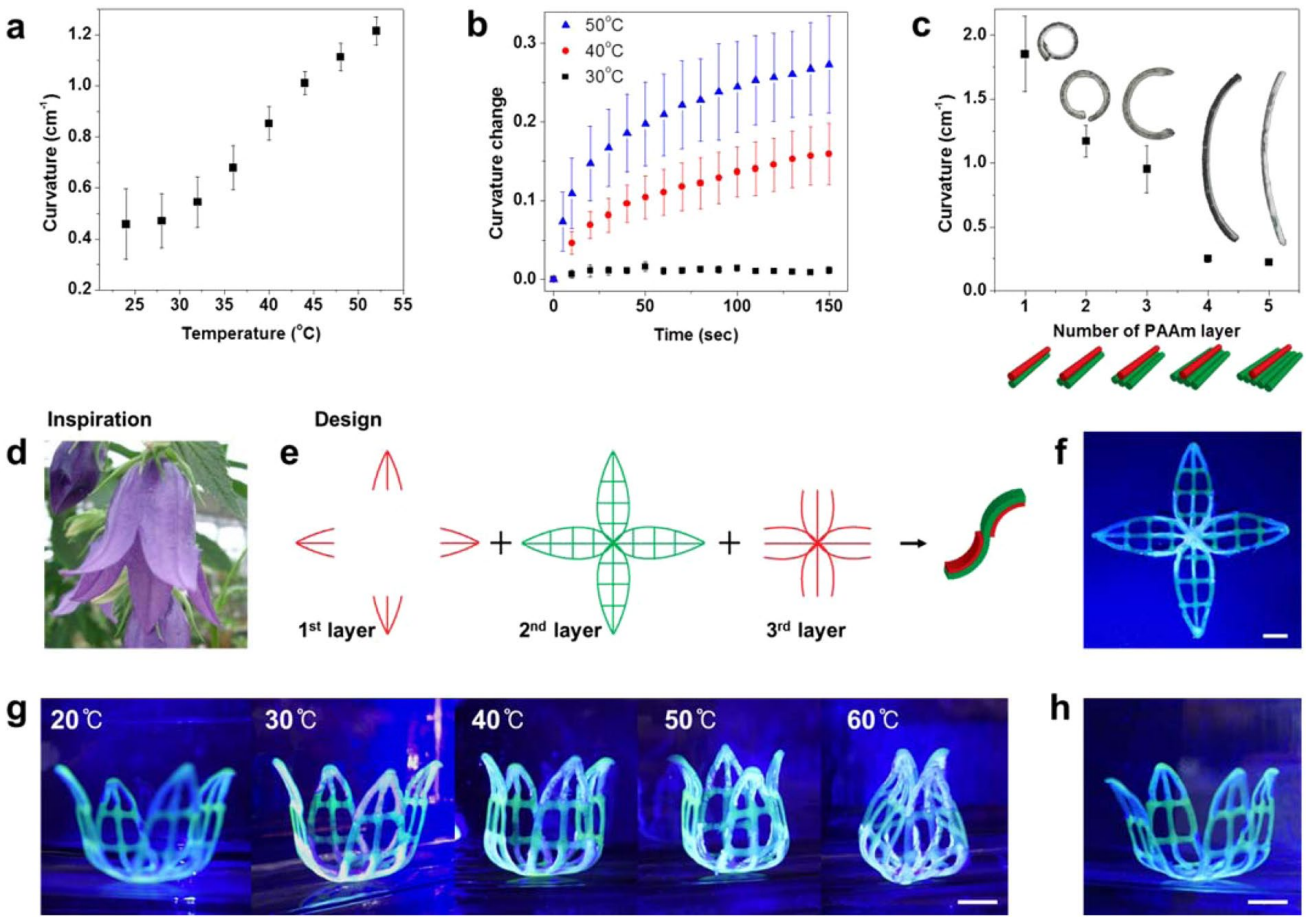
Characterization of the bending behaviors of printed bilayers consisting of PNIPAAm and PAAm hydrogels (with a constant length of 5 cm, printed with an 800-μm nozzle) and predicted 4D printing of biomimetic architectures. The inks used here were formulated with the composition of 200 wt% of sucrose with 60 wt% of glucose. (**a**) Bending curvature versus temperature in water. **(b**) Changed curvature versus immersion time in water at various temperatures. (**c**) Bending curvature as a function of the number of passive PAAm layers (green) at 40 °C. (**d**) A bellflower, the inspiration for the 4D printing experiment. (**e)** 3D printing design based on the bellflower. Red is PNIPAAm, and green is PAAm. (**f**) Final printed structure. (**g**) Temperature-induced shape changes in the printed design. (h) Recovered shape after the structure had been cooled to 20 °C. Scale bars in (**f-h**) are 1 cm. Error bars in (**a–c**) represent the standard deviations of five experiments.

To investigate the bending behavior of the printed architectures, we characterized the influence of the temperature and the thickness of the hydrogel (Fig. 4a–c). The temperature-induced bending curvatures of 5-cm-long printed bilayers of PNIPAAm and PAAm were measured. Immersing the bilayers in hot water led to a change in their curvature owing to asymmetric strain induced by the substantially larger amount of water expulsion from the PNIPAAm hydrogel^7–9^. The bending curvature and the bending rate increased with increasing temperature (Fig. 4a,b). Compared with the conventional PNIPAAm hydrogel, the macroporous hydrogel showed a faster response to temperature change. Whereas conventional hydrogels required tens of minutes to hours to change shape^7–9,12,13^, our macroporous hydrogels changed shape within only a few seconds to minutes. The dimensions of each hydrogel layer in a multilayer structure can, thus, be adapted to trigger and control its bending precisely. Printing was repeated to vary the width of the passive (PAAm) layer underneath the PNIPAAm layer. The bending curvature decreased as the width of the passive layer (PAAm) increased (Fig. 4c). Thus, the shape-morphing properties of the printed architectures could be controlled by changing the external temperature and the thickness of the printing design.

As an example of 4D printing of hydrogel architectures, we created a printed structure that mimicked the shape and petal movements of a bellflower, which has multiple curvatures (Fig. 4d). The printing design called for three different layers (Fig. 4e) to mimic the S-shape curvatures of the bellflower’s petals because the structure curves toward the PNIPAAm layers. The resulting printed pattern (Fig. 4f) was immersed in water to initiate swelling and shape morphing in response to temperature changes (Fig. 4g). The printed pattern, which was initially flat-printed, folded into a flower shape, and the curvature of the “petals” increased with increasing temperature. As previously explained, because this architecture was composed of macroporous hydrogels, the total shape-morphing process of the printed structure was faster than that of conventional PNIPAAm hydrogels (Movie S1, Supporting Information). The process of unfolding the structure, caused by swelling of the PNIPAAm layer in response to decreasing temperature, was slower than the folding process. We should point out that the shape morphing during temperature rise could be measured systematically with accurate temperature control. However, the shape morphing was irregular and inconsistent during the recovery process of cooling. In most cases, the structure fully recovered its initial shape,which ccurred at 20 °C after 15 min (Fig. 4h).

## Conclusion

In summary, we fabricated sugar/hydrogel inks for 3D printing and used them to print multilayered architectures that demonstrated temperature-induced shape morphing. Temperature-sensitive PNIPAAm hydrogel was used as the active layer, and PAAm was used as the passive layer. Sugars added to the inks increased their yield stress and led to macropore formation, which in turn enabled fast responses in the printed architectures. This ink fabrication and printing technique is easy to perform, does not require organic solvents, and is widely applicable to various materials. The sugar-enhanced hydrogel ink can also be used to print biocompatible materials, such as gelatin methacryloyl and other stimuli-responsive materials, such as poly(acrylic acid). Four-dimensional printing through control of the printing parameters (i.e., thickness and orientation) and the temperature of the immersion water enabled fabrication of multilayered architectures with programmable morphing into a targeted shape. By using representative examples of PNIPAAm and PAAm, varying the thickness of each layer, and varying the temperature, we were able to vary the speed and the extent of shape morphing. Our flexible ink design, thus, can provide a new fabrication method that can open new avenues for creating programmable shape-morphing architectures in various applications, such as soft robotics and tissue engineering.

## Experimental Methods

### Preparation of sugar/hydrogel ink

For each ink, monomers (3-M NIPAAm or 4-M AAm) were dissolved in deionized water. *N*,*N*′-Methylenebisacrylamide (MBAA) was added to the aqueous monomer solution as a chemical cross-linker. Analytical-grade ground sucrose of 200 wt% and glucose of 60 wt% relative to the amount of solvent were added to the solutions with vigorous stirring at 60 °C to melt the sugars. A photoinitiator, (2-hydroxy-2-methylpropiophenone, 90 mM) and a medium-viscosity alginic acid sodium salt (30 wt% of AAm and 20 wt% of NIPAAm) were added to the solutions. All chemicals were purchased from Sigma-Aldrich.

### Characterization of sugar/hydrogel ink

The rheological properties of the sugar/hydrogel inks were characterized at 25 °C by using a controlled stress rheometer (Discovery HR-1 hybrid rheometer, TA Instruments). A 40-mm plate (0.4-mm measuring gap) was used for the monomer ink, and a 25-mm plate (1-mm measuring gap) was used for alginate ink and sugar ink. Flow sweeps were performed at speed between 0.1 and 1300 s^−1^. Oscillatory measurements were performed at an angular frequency of 10 rad s^−1^ for pressures between 0.1 and 10,000 Pa.

### Characterization of macroporous hydrogels

In experiments involving morphological testing of gels (not prints), all samples were prepared in 1-M NIPAAm for clear observation. For each sample, 1-M NIPAAm and 20-mM MBAA were dissolved in deionized water. Sucrose with a certain concentration (wt% relative to the solvent) was added to study the effect of sugar on the porosity of the gel. The photoinitiator (30 mM) and the alginic acid sodium salt (20 wt%) were added to the aforementioned solutions. Pre-gel solutions were exposed to UV light for 30 min to induce polymerization, and the resulting hydrogels were immersed in a 10% (w/v) CaCl_2_ solution.

Hydrogels were swollen to equilibrium at room temperature for at least 24 h with the water changed every 6 h; the hydrogels were subsequently freeze-dried in a freeze dryer (FDU-1200, EYELA) for at least 24 h to remove the water completely. After drying, the gel samples were sputter coated with gold and then visualized using a scanning electron microscope (JSM-7100F, Jeol). The relative pore area of the sample was measured using the ImageJ program (National Institutes of Health).

The mass swelling ratios (*R*_*m*_) of the hydrogels with different sucrose concentrations are given by *M*_*w*_/*M*_*d*_, where *M*_*w*_ is the weight of a swollen hydrogel and *M*_*d*_ is the weight of the hydrogel after it has been dried in an oven at 60 °C for at least 24 h. The *M*_*w*_ was measured gravimetrically after the gel had been incubated in deionized water for at least 24 h at 25 °C and the excess surface water blotted with moistened filter paper.

To investigate the dependence of the hydrogels’ shrinkage dynamics on their macroporosity, we defined the dynamic thermoresponsive shrinking ratio of PNIPAAm hydrogels (*R*_*t*_)_25→50_ as *A*_*t*_/*A*_0_, where *A*_*t*_ is the area (cm^2^) of a hydrogel at *t* seconds and *A*_0_ is the area (cm^2^) of the hydrogel at 0 s. Sample hydrogels were prepared to a thickness of approximately 1 mm in 35-mm round Petri dishes. Before the experiment, the hydrogels were allowed to swell to equilibrium in deionized water at 25 °C for at least 24 h. The swollen gels were then transferred into a water bath at 50 °C for measuring the shrinkage at different times. Parameter *A*_0_ is the area (cm^2^) of the initial swelling state before the hydrogel was transferred to the 50 °C bath. Temperature control of the water was carried out using a water bath (WiseBath, Wisd Co.).

### Three-dimensional (3D) printing procedure

Computer-aided design (CAD) models were developed using AutoCAD 2016 (Autodesk) and exported as stereolithography (STL) data. The STL data were imported into the Simplify3D (Simplify3D LLC) software, which converted it to print paths via G-codes. The printed samples were fabricated layer by layer according to the G-code instructions by using a DeltaBot printer (lab311, Korea) and a BIO X (Cellink, Sweden). The inks were loaded into a 50-mL syringe and deposited using a pneumatic pump through tapered nozzles with diameters of 600 µm and 800 µm under pressures ranging from 60 to 100 kPa. The printing speed was 10 cm s^−1^.

### Synthesis of macroporous hydrogels

Immediately after printing, the samples were cured for 1–5 min by using a UV source (8 W, 254 nm, VL-8.MC, Vilber Lourmat). After curing, the samples were immersed in a 10% (w/v) CaCl_2_ (aq) solution for simultaneous ionic cross-linking of alginate and leaching of sugar.

### Characterization of bending behaviors

Samples with a 5-cm bilayer design were printed, as described above, by using the 800-µm-diameter nozzle. The samples were immersed in a water bath for precise temperature control. The bending curvature of the samples was measured from videos recorded using an iPhone 6 S. Images extracted from the video were analyzed with the ImageJ program to determine the bending curvature of the samples. Shape-morphing motions of the samples in the water bath were recorded under LED UV flashlight (3 W, 390–395 nm).

## Supplementary information

Movie S1.

Figure S1 and Figure S2.

## References

[1] Kang H-W (2016). A 3D bioprinting system to produce human-scale tissue constructs with structural integrity. Nature Biotechnology.

[2] Tamayol A (2015). Bioactive Fibers: Hydrogel Templates for Rapid Manufacturing of Bioactive Fibers and 3D Constructs (Adv. Healthcare Mater. 14/2015). Advanced Healthcare Materials.

[3] Truby RL, Lewis JA (2016). Printing soft matter in three dimensions. Nature.

[4] Roh S, Parekh DP, Bharti B, Stoyanov SD, Velev OD (2017). 3D Printing by Multiphase Silicone/Water Capillary Inks. Advanced Materials.

[5] Valentine AD (2017). Hybrid 3D Printing of Soft Electronics. Advanced Materials.

[6] Farahani RD, Dubé M, Therriault D (2016). Three-Dimensional Printing of Multifunctional Nanocomposites: Manufacturing Techniques and Applications. Advanced Materials.

[7] Cao T (2017). Chelator-Free Conjugation of 99mTc and Gd3 to PEGylated Nanographene Oxide for Dual-Modality SPECT/MR Imaging of Lymph Nodes. ACS Applied Materials & Interfaces.

[8] Palleau, E., Morales, D., Dickey, M. D. & Velev, O. D. Reversible patterning and actuation of hydrogels by electrically assisted ionoprinting. *Nature Communications* **4**, 10.1038/ncomms3257 (2013). 10.1038/ncomms3257 23907294

[9] Ionov L (2015). Polymeric Actuators. Langmuir.

[10] Deng J (2016). Tunable Photothermal Actuators Based on a Pre-programmed Aligned Nanostructure. Journal of the American Chemical Society.

[11] Hines L, Petersen K, Lum GZ, Sitti M (2017). Soft Actuators for Small-Scale Robotics. Advanced Materials.

[12] Gladman AS (2016). Biomimetic 4D printing. Nature Materials.

[13] Raviv, D. *et al*. Active Printed Materials for Complex Self-Evolving Deformations. *Scientific Reports* **4**, 10.1038/srep07422 (2015). 10.1038/srep07422 PMC4270353 25522053

[14] Ge, Q. *et al*. Multimaterial 4D Printing with Tailorable Shape Memory Polymers. *Scientific Reports* **6**, 10.1038/srep31110 (2016). 10.1038/srep31110 PMC4976324 27499417

[15] Li Y-C, Zhang YS, Akpek A, Shin SR, Khademhosseini A (2016). 4D bioprinting: the next-generation technology for biofabrication enabled by stimuli-responsive materials. Biofabrication.

[16] Forterre Y, Skotheim JM, Dumais J, Mahadevan L (2005). How the Venus flytrap snaps. Nature.

[17] Peng X, Wang H (2018). Shape Changing Hydrogels and Their Applications as Soft Actuators. J. Polym. Sci. Pol. Phys.

[18] Pei E, Loh GH (2018). Technological considerations for 4D printing: an overview. Prog. Addit. Manuf.

[19] Jia H (2019). Universal Soft Robotic Microgripper. Small.

[20] Wang E, Desai MS, Lee S-W (2013). Light-Controlled Graphene-Elastin Composite Hydrogel Actuators. Nano. Lett..

[21] Mou C-L (2014). Monodisperse and Fast-Responsive Poly(N-isopropylacrylamide) Microgels with Open-Celled Porous Structure. Langmuir.

[22] Zhang J-T, Cheng S-X, Huang S-W, Zhuo R-X (2003). Temperature-Sensitive Poly(N-isopropylacrylamide) Hydrogels with Macroporous Structure and Fast Response Rate. Macromolecular Rapid Communications.

[23] Zhang J-T, Cheng S-X, Zhuo R-X (2003). Preparation of macroporous poly(N-isopropylacrylamide) hydrogel with improved temperature sensitivity. Journal of Polymer Science Part A: Polymer Chemistry.

[24] Maeda S, Kato T, Kogure H, Hosoya N (2015). Rapid response of thermo-sensitive hydrogels with porous structures. Applied Physics Letters.

[25] Zhang, X.-Z. & Chu, C.-C. Thermosensitive PNIPAAm cryogel with superfast and stable oscillatory properties. *Chemical Communications* 1446–1447, 10.1039/b301423a (2003). 10.1039/b301423a 12841284

[26] Chatterjee P, Dai A, Yu H, Jiang H, Dai LL (2015). Thermal and mechanical properties of poly(N-isopropylacrylamide)-based hydrogels as a function of porosity and medium change. Journal of Applied Polymer Science.

[27] Park JH (2010). Microporous cell-laden hydrogels for engineered tissue constructs. Biotechnology and Bioengineering.

[28] Hong S (2015). 3D Printing: 3D Printing of Highly Stretchable and Tough Hydrogels into Complex, Cellularized Structures. Advanced Materials.

[29] Haq MA, Su Y, Wang. D (2017). Mechanical properties of PNIPAM based hydrogels: A review. Materials Science and Engineering C.

[30] Drury JL, Dennis RG, Mooney DJ (2004). The tensile properties of alginate hydrogels. Biomaterials.

[31] Wang MX (2015). Tough Photoluminescent Hydrogels Doped with Lanthanide. Macromolecular Rapid Communications.

[32] Sun J-Y (2012). Highly stretchable and tough hydrogels. Nature.

[33] Malda J (2013). 25th Anniversary Article: Engineering Hydrogels for Biofabrication. Advanced Materials.

[34] Smith PT, Basu A, Saha A, Nelson A (2018). Chemical modification and printability of shear-thinning hydrogel inks for direct-write 3D printing. Polymer.

[35] Ribeiro, A. C. F *et al*. Diffusion coefficient and electrical conductivities for calcium chloride aqueous solutions at 298.15 K and 310.15 K, *Electrochimica Acta* **54**, 192–196,

[36] Linder, P. W. et al. The diffusion coefficient of sucrose in water. A physical chemistry experiment. *Journal of Chemical Education* **53**, 330–332, 10.1021/ed053p330 (1976), 10.1016/j.electacta.2008.08.011 (2008).

[37] Caliari SR, Burdick JA (2016). A practical guide to hydrogels for cell culture. Nature Methods.

[38] Otake K, Inomata H, Konno M, Saito S (1990). Thermal analysis of the volume phase transition with N-isopropylacrylamide gels. Macromolecules.

