# Supplementary material for: Formulation of Sugar/Hydrogel Inks for Rapid Thermal Response 4D Architectures with Sugar-derived Macropores
Source: Sci Rep. 2020 May 5;10:7527. doi: 10.1038/s41598-020-64457-8 (PMC7200689; doi:10.1038/s41598-020-64457-8)
Supplement: Supplementary file 2 — Figure S1 and Figure S2. [file 41598_2020_64457_MOESM2_ESM.docx]

Supplementary Information

Formulation of Sugar/Hydrogel Inks for Rapid Thermal Response 4D Architectures with Sugar-Derived Macropores

**Hyojin Ko^1^, Monica Cahyaning Ratri^1,2^, Kihoon Kim^1^, Yeongheon Jung^1^, Giyoong Tae^3^, and Kwanwoo Shin^1,*^**

^1^Department of Chemistry and Institute of Biological Interfaces, Sogang University, Seoul 04107, Republic of Korea

^2^Department of Chemistry Education, Sanata Dharma University, Yogyakarta 55281, Republic of Indonesia

^3^School of Materials Science and Engineering, Gwangju Institute of Science and Technology, Gwangju 61005, Republic of Korea

**Movie S1.** Video of a programmed, reshaping hydrogel architecture.


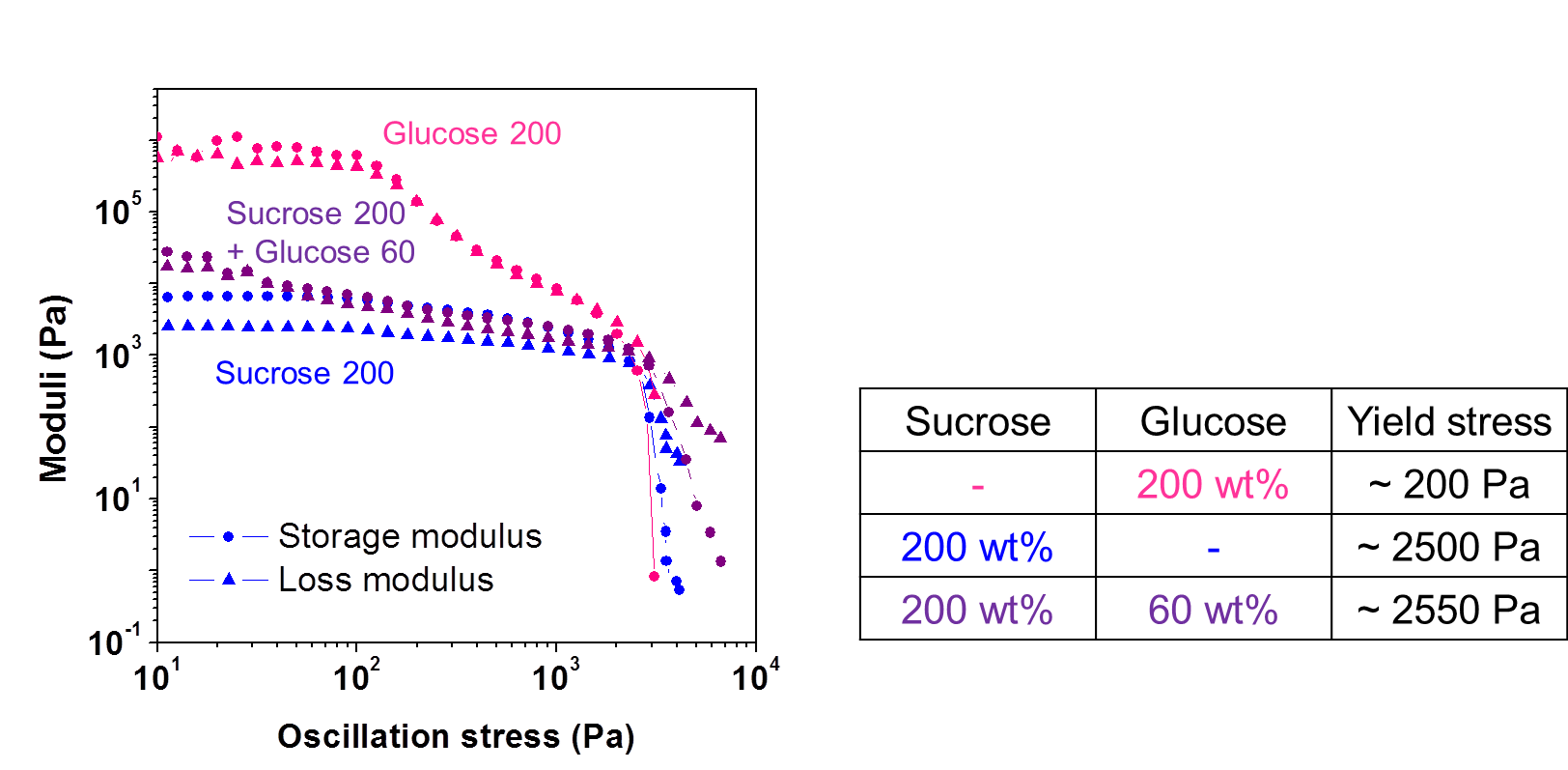


**Figure S1.** Storage and loss moduli as functions of the oscillation stress for inks with different sucrose and glucose concentrations.


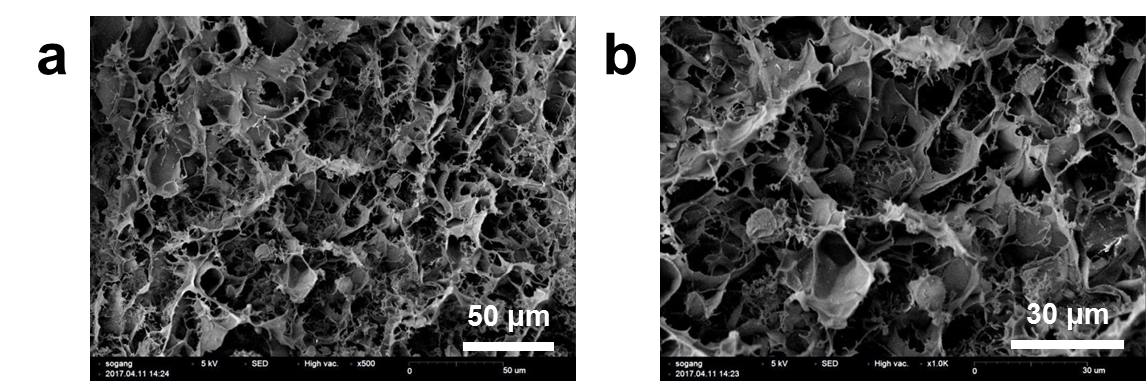


**Figure S2.** (a) SEM image of 3-M PNIPAAm hydrogels with 200 wt% of sucrose. (b) A higher-magnification view of the image in (a).
